# Evaluation of the effects of black soldier fly larval hydrolysate on layer chicks

**DOI:** 10.1016/j.psj.2026.107676

**Published:** 2026-08-20

**Authors:** Xinhao Zhou, Wenye Zhang, Shuaichen Yao, Yaxin Pei, Jiran Zhang, Xiaojun Feng, Sen Yang

**Affiliations:** a College of Life Sciences, Henan Agricultural University, Zhengzhou, 450046, China; b College of Life Science and Technology, Huazhong Agricultural University, Wuhan, 430070, China; c Lingbao City Agricultural and Rural Bureau, Lingbao, 472500, China

**Keywords:** Black soldier fly larval hydrolysate, Layer chicks, Growth performance, Intestinal health, Antioxidant capacity

## Abstract

This study investigated the effects of dietary supplementation with black soldier fly larval hydrolysate (**BSFH**), an un-defatted crude hydrolysate, on the growth performance, immune organ indices, serum biochemical parameters, antioxidant status, intestinal morphology, and cecal microbiota of layer chicks. A total of 360 one-day-old Gushi layer chicks were randomly allocated to four treatment groups: a control group fed a corn-soybean meal basal diet and three experimental groups (**E3, E6**, and **E9**) supplemented with 0.3%, 0.6%, and 0.9% BSFH, respectively. The experimental period lasted for 63 days. Results indicated that BSFH supplementation dose-dependently improved average daily gain and feed-to-gain ratio. Average daily gain was significantly increased at the 0.6% and 0.9% levels, whereas feed-to-gain ratio was significantly improved only at the 0.9% level, with the 0.3% and 0.6% levels showing numerical trends; average daily feed intake was not significantly affected. BSFH supplementation did not significantly affect spleen or Fabricius bursa indices. At the serum biochemical level, BSFH at 0.3% and 0.6% significantly reduced alanine aminotransferase activity, while uric acid levels were dose-dependently decreased, with significant reductions observed at 0.6% and 0.9% supplementation levels; serum albumin was significantly increased at the 0.3% and 0.6% levels, and high-density lipoprotein cholesterol showed a linear increasing trend with supplementation level. BSFH also enhanced antioxidant enzyme activities, with serum glutathione peroxidase and catalase peaking at the 0.3% level, serum total superoxide dismutase peaking at the 0.6% level, and hepatic catalase being highest at the 0.6% level. Intestinal morphology analysis revealed that BSFH reduced jejunal crypt depth and increased the villus height to crypt depth ratio, with these effects observed specifically in the jejunum, while no significant changes were detected in the duodenum or ileum. Cecal microbiota analysis indicated that BSFH did not significantly alter overall microbial diversity but enriched the genus *Fusobacterium* in the cecum, with the highest relative abundance observed at the 0.6% supplementation level; species-level sequencing is warranted to clarify the functional implications of this shift. These findings suggest that BSFH is a promising and sustainable protein source for poultry feed, capable of promoting growth and potentially improving the overall health status of layer chicks.

## Introduction

The global poultry industry faces escalating challenges, necessitating the development of sustainable and functionally effective protein sources for feed. Traditional high-protein ingredients, such as fish meal and soybean meal, are increasingly constrained by supply shortages, price volatility, and anti-nutritional factors, making it difficult to meet the demands of a rapidly expanding industry. Consequently, the search for effective alternatives has become increasingly urgent (David et al., 2024; Lanza et al., 2025; Keohavong, 2026). In this context, the black soldier fly (*Hermetia illucens*) has emerged as a promising protein alternative, owing to its abundant resource base, rapid growth cycle, environmentally friendly rearing characteristics, and the inherent bioactive properties of its products (Sajid et al., 2023).

Black soldier fly larvae (**BSFL**) are rich in high-quality protein, unique fatty acids, and chitin, among other bioactive components (Vázquez et al., 2024; Warisman et al., 2024; Gadzama, 2025). These constituents collectively contribute to a balanced nutritional profile and diverse functional properties (Fruci et al., 2023; Silva et al., 2024). Existing research has highlighted the broad application prospects of black soldier fly products in improving animal growth performance, promoting gut health, enhancing antioxidant capacity, and modulating inflammatory responses (Li et al., 2023, 2024). However, some studies have also indicated that at high inclusion levels, black soldier fly products may exert adverse effects on feed intake or production performance in animals (Fruci et al., 2023).

To enhance the bioavailability of insect protein and mitigate its antinutritional factors, enzymatic hydrolysis has emerged as an effective processing strategy. In this study, black soldier fly larvae were treated by whole-body enzymatic hydrolysis, primarily targeting the protein fraction. This process yields a hydrolysate containing small molecular peptides, free amino acids, residual lipids, and minerals, thereby preserving the functional constituents of the whole larvae. Protein hydrolysates are rich in small molecular peptides and free amino acids, which not only substantially improve protein digestion and absorption rates but may also confer enhanced bioactive properties, including antioxidant and immunomodulatory activities (Hall et al., 2018; Mikhailova et al., 2024; Muñoz-Seijas et al., 2024). Previous studies have demonstrated that BSFL protein hydrolysate supplementation improves palatability in cats (Li et al., 2024) and enhances antioxidant and anti-inflammatory capacity in dogs (Wei et al., 2024). In poultry models, protein hydrolysates from various sources have been shown to effectively improve growth performance, optimize intestinal morphology, and modulate gut microbial balance (Tolba et al., 2023; Zhang et al., 2024).

Layer chicks are in a critical phase of rapid growth and development, characterized by high metabolic activity and heightened sensitivity to nutritional supply and environmental stressors. Although existing evidence indicates that dietary supplementation with full-fat or defatted black soldier fly larval meal (**BSFLM**) enhances productive performance (Chen et al., 2025), serum biochemical indices, and antioxidant capacity (Li et al., 2025), as well as modulates cecal microbiota composition, it remains unclear whether BSFH, as a potentially more bioactive form, can exert comparable or superior effects on growth performance, serum biochemical parameters, immune function, antioxidant status, intestinal morphology, and cecal microbiota in layer chicks. Therefore, this study was conducted to comprehensively evaluate the effects of dietary supplementation with graded levels of BSFH on productive performance, immune organ indices, serum biochemical parameters, antioxidant status, intestinal morphology, and cecal microbial community in layer chicks. The findings of this study are expected to provide a scientific foundation for the application of BSFH as a sustainable and functional feed ingredient in poultry diets.

## Material and methods

### Birds, housing and experimental diets

A total of 360 one-day-old Gushi G-line layer chicks, showing good health and similar body weights (average 32.79±0.4 g), were selected for this experiment. The chicks were randomly assigned to four treatment groups, each comprising six replicates with 15 birds per replicate. The control group received a corn-soybean meal basal diet, whereas experimental groups E3, E6, and E9 were fed the basal diet supplemented with 0.3%, 0.6%, and 0.9% BSFH, respectively. The experimental diets were not formulated to be strictly isonitrogenous and isoenergetic. BSFH was supplemented as a feed additive on top of the basal diet, without replacing any basal ingredient. The ingredient composition and nutrient contents of the basal diet are presented in Table 1, while the nutritional components and hygiene indicators of BSFH are detailed in Table 2. The BSFH was prepared from frozen fifth-instar black soldier fly larvae. Briefly, thawed larvae were mixed with a citric acid solution at a ratio of 1:0.6 (larvae : citric acid solution, w/w), and the pH was adjusted to 5.5. Hydrolysis was subsequently performed with 0.1% (w/w) acidic protease (Ningxia Sunson Industrial Group Co., Ltd.; activity: 100,000 U/g) at 45°C for 72 h. The hydrolysate was then inactivated, filtered, and dried to obtain the final BSFH product. A 7-day pre-experimental period was followed by a 56-day experimental period.

**Table 1:** Composition and nutrient levels of basal diets.

Items	Value
Ingredients,(%)	
Corn,(%)	65.55
Soybean meal,(%)	26.00
Wheat bran,(%)	2.80
Soybean oil,(%)	1.50
Limestone,(%)	1.20
Salt,(%)	0.30
*DL*-Met,(%)	0.10
*L*-Lys·HCl,(%)	0.05
Dicalcium phosphate,(%)	1.50
Premix^1^	1.00
Total,(%)	100
Nutrient levels^2^	
ME(MJ/kg)^2^	12.26
Cp,(%)	17.43
Ca,(%)	0.93
P,(%)	0.69
AP,(%)	0.37
Lys,(%)	0.92
Met,(%)	0.38
Met+Cys,(%)	0.68

1 The premix provided the following per kg of diet: VA 15000 IU, VD_3_ 3000 IU, VE 60 mg, VK_3_ 10 mg, VB_1_ 3 mg, VB_2_ 9 mg, VB_6_ 7 mg, VB_12_ 0.04 mg, biotin 0.12 mg, folic acid 1.5 mg, Pantothenic acid 15 mg, nicotinic acid 50 mg, Cu (as copper sulfate) 8 mg, Fe (as ferrous sulfate) 80 mg, Mn (as manganese sulfate) 60 mg, Zn (as zinc sulfate) 40 mg, I (as potassium iodide) 0.35 mg, Se (as sodium selenite) 0.15 mg.

2 ME was a calculated value, while the others were measured values.

**Table 2:** Nutritional components and hygiene indicators of BSFH.

Indicators	Content
Crude Protein,(%)	41.17
Crude Fat,(%)	23.2
Crude Ash,(%)	14.2
Calcium,(%)	3.98
Phosphorus,(%)	0.95
Peptide content (less than 3 KDa),(%)	39.31

All layer chicks were housed in the same poultry house at the Poultry Breeding Experimental Laboratory, Henan Agricultural University Science and Education Park. A four-tier stepped cage system was used. Each cage measured 120 cm (length) × 60 cm (width) × 38 cm (height), with 15 birds per cage. Two end-type nipple drinkers and one round-type galvanized tube feeder were installed per cage, allowing ad libitum access to feed and water throughout the 63-day experimental period. Feed was provided manually twice daily, at 08:30 and 14:30. A standard immunization program was implemented, including Newcastle disease (ND) vaccine and infectious bronchitis (IB) vaccine (eye drop, day 1), ND LaSota booster (drinking water, day 7), infectious bursal disease (IBD) vaccine (drinking water, day 14), ND LaSota booster (drinking water, day 21), fowlpox (FP) vaccine (wing-web stab, day 28), and IBD vaccine booster (drinking water, day 35), following the routine protocol for commercial layer flocks in Henan Province. The health status of the chicks was closely monitored, and feces and waste were promptly removed from the cages to prevent disease transmission. This experiment was conducted in accordance with the Guidelines for Ethical Review of Experimental Animals and approved by the Animal Care and Use Committee of Henan Agricultural University (Approval No. 25-0146).

### Performance

Daily feed intake and residual feed were recorded for each replicate. Residual feed was collected and weighed each morning at 08:00 prior to feeding. Feed intake was calculated as the difference between the amount of feed provided and the residual feed remaining. Initial body weight of chicks was measured on day 1 of the formal experiment (at approximately 08:00, before feeding), and individual body weights were subsequently measured weekly at the same time of day. At the end of the experiment (day 63), following an overnight 12-h fasting period, final body weights were recorded at 08:00. All body weight and feed measurements were performed using an electronic precision balance (Shanghai Liangping YP-10002; readability: 0.01 g). Average daily gain (**ADG**), average daily feed intake (**ADFI)**, and feed-to-gain ratio (**F/G**) were calculated as follows:

ADG(g/bird/day)=(Finalbodyweight−Initialbodyweight)/Experimentaldays

ADFI(g/bird/day)=Totalfeedintakeperreplicate/(Numberofbirdsperreplicate×Experimentaldays)

F/G=ADFI/ADG

### Immune organ index

At the end of the experiment, following a 12 h fasting period, one healthy chick was randomly selected from each replicate and weighed to record its live weight. The spleen and bursa of Fabricius, as primary immune organs, were then carefully excised. Adipose tissue was removed from these organs, and residual blood was blotted dry before weighing. Immune organ indices were calculated as the ratio of organ weight to live body weight.

### Serum biochemical profile

At the end of the experiment, one chick was randomly selected from each replicate for blood sampling. After a 12 h fasting period, 5 mL of blood was collected from the wing vein. Samples were allowed to clot for 30 minutes at room temperature, then centrifuged at 3,000 rpm for 15 minutes. The supernatant serum was carefully transferred to clean centrifuge tubes and stored at −80°C until analysis. Serum levels of alanine aminotransferase (**ALT**), aspartate aminotransferase (**AST**), alkaline phosphatase (**ALP**), albumin (**ALB**), urea (**UREA**), uric acid (**UA**), total bile acid (**TBA**), triglycerides (**TG**), total cholesterol (**CHO**), high-density lipoprotein cholesterol (**HDL-C**), and low-density lipoprotein cholesterol (**LDL-C**) were determined using an automated biochemical analyzer (BS-240VET).

### Antioxidant parameters

Following blood collection, chicks were euthanized by exsanguination and dissected. Approximately 0.5 g of liver tissue was collected, placed in self-sealing bags, and stored at −80°C. Commercial assay kits (Servicebio Co., Ltd.) were used to determine the levels of glutathione peroxidase (**GSH-Px**, G4305), total superoxide dismutase (**T-SOD**, G4306), catalase (**CAT**, G4307), total antioxidant capacity (**T-AOC**, G4313), and malondialdehyde (**MDA**, G4302) in both serum and liver samples.

### Intestinal morphology

At the conclusion of the experiment, one chick was randomly selected from each replicate. After a 12 h fasting period, intestinal samples were collected. Approximately 3 cm segments of the duodenum, jejunum, and ileum were excised and immediately fixed in 4% paraformaldehyde solution. After fixation for more than 24 h, samples underwent routine paraffin embedding, sectioning, and hematoxylin-eosin (**H.E.**) staining. Sections were then mounted with neutral gum and observed under an optical microscope. Villus height (**V**) and crypt depth (**C**) were measured, and the villus height-to-crypt depth ratio (**V/C**) was calculated.

### Cecal microbiota

For cecal microbiota analysis, five chicks (one chick per replicate, with one randomly omitted replicate per treatment group) were randomly selected at slaughter, and cecal contents were rapidly collected into 1.5 mL centrifuge tubes and stored at −80°C. Total DNA was extracted from the cecal contents. The extracted genomic DNA was verified by 1% agarose gel electrophoresis. Specific primers 338F_806R (338F: ACTCCTACGGGAGGCAGCAG; 806R: GGACTACHVGGGTWTCTAAT) with barcodes were synthesized for PCR amplification targeting the V3-V4 region of the 16S rRNA gene. PCR products from the same sample were pooled and verified by 2% agarose gel electrophoresis. PCR products were purified using an AxyPrep DNA Gel Extraction Kit. Sequencing was performed on the Illumina MiSeq PE300 platform by Majorbio Bio-Pharm Technology Co. (Shanghai, China) to obtain valid data for analyzing cecal microbial community composition.

### Statistical analysis

Experimental data were analyzed using IBM SPSS Statistics 26.0 software. One-way analysis of variance (**ANOVA**) was performed, followed by Duncan's multiple comparison test. Polynomial contrasts (linear and quadratic) were used to assess the effects of different levels of BSFH on chicks. Results are presented as mean ± standard deviation (**SD**) for 6 replicate samples, with the exception of cecal microbiota analysis, where n = 5 per group, along with their pooled standard error of the mean (**SEM**). Statistical significance was set at *P* < 0.05, indicated by different lowercase letters. Origin 2024 software was used for plotting figures.

## Results

### Performance of layer chicks fed BSFH

The effects of dietary supplementation with graded levels of BSFH on the growth performance of layer chicks were evaluated, and the results are presented in Table 3. Compared with the control group, supplementation with 0.3%∼0.9% hydrolysate did not significantly affect the average daily feed intake of the chicks, although the feed intake in all experimental groups was numerically higher than that of the control group. This indicates that the hydrolysate exhibits good palatability and is compatible with the basal diet without causing feed aversion in chicks. Compared with the control group, the average daily weight gain of the chicks in the E3, E6 and E9 groups increased by 3.68% (*P* = 0.055), 5.49% (*P* = 0.008), and 8.01% (*P* < 0.001), respectively. Statistical analysis revealed that ADG showed a significant linear (*P* < 0.001) and quadratic (*P* = 0.001) effect with the increase in BSFH addition, indicating that BSFH can effectively promote the growth of chicks. The feed-to-gain ratios of the chicks in the E6 and E9 groups decreased by 3.49% (*P* = 0.059) and 6.20% (*P* = 0.004) respectively, demonstrating significant linear (*P* = 0.001) and quadratic (*P* = 0.006) effects as well. These results demonstrate that supplementation with the hydrolysate effectively promotes chick growth and improves feed conversion efficiency. The daily gain increased with the supplementation level, exhibiting a clear dose-dependent trend. The concomitant improvement in feed conversion efficiency further suggests that the growth-promoting effect was not solely attributable to increased feed intake but largely resulted from enhanced nutrient utilization efficiency, thereby optimizing the growth conversion process without increasing feed intake burden.

**Table 3:** Effects of BSFH with different addition amounts on the production performance of layer chicks.

Item	Group				SEM	*p-*Value		
	Control	E3	E6	E9		ANOVA	Linear	Quadratic
Average initial weight/g	32.62±0.54	32.62±0.52	32.86±0.19	33.08±0.14	0.10	0.319	0.068	0.170
Average terminal weight/g	900.25±35.34^c^	932.00±9.06^bc^	947.75±4.35^ab^	969.75±20.84^a^	8.06	0.004	<0.001	0.001
ADG(g·d^−1^)	15.49±0.63^c^	16.06±0.16^bc^	16.34±0.08^ab^	16.73±0.37^a^	0.14	0.004	<0.001	0.001
ADFI(g•d^−1^)	39.98±0.78	40.93±1.48	40.61±0.55	40.44±1.02	0.26	0.659	0.655	0.525
F/G	2.58±0.08^a^	2.55±0.08^a^	2.49±0.05^ab^	2.42±0.09^b^	0.02	0.019	0.001	0.006

Note: In the same row, values with the same letter mean no significant difference (*p* > 0.05), while with different letter mean significant difference (*p* < 0.05). The same as below.

### Immune organ index of layer chicks fed BSFH

As shown in Table 4, BSFH supplementation did not significantly affect spleen index or bursa index in layer chicks (P > 0.05). Although numerical increases of 8.59%, 19.19%, and 24.75% in spleen index, and 8.12%, 15.81%, and 26.07% in bursa index were observed in the E3, E6, and E9 groups, respectively, compared with the control, these differences did not reach statistical significance (*P* > 0.05).

**Table 4:** Effects of BSFH with different addition amounts on the immune organ index of layer chicks.

Item	Group				SEM	*p-*Value		
	Control	E3	E6	E9		ANOVA	Linear	Quadratic
Spleen index	1.98±0.61	2.15±0.19	2.36±0.63	2.47±0.30	0.11	0.393	0.075	0.213
Fabricius index	2.34±0.51	2.53±0.26	2.71±0.41	2.95±0.74	0.12	0.332	0.058	0.172

### Serum biochemical profile of layer chicks fed BSFH

Based on the determination of serum biochemical indicators in layer chicks fed diets supplemented with graded levels of BSFH, this section systematically analyzes its effects on protein metabolism, liver function, and lipid metabolism in chicks, evaluating its potential physiological regulatory role from a blood biochemical perspective. The results are presented in Table 5. Regarding liver function, serum ALT activity in the E3 and E6 groups was significantly lower than that in the control group, with reductions of 49.86% (*P* = 0.008) and 45.04% (*P* = 0.013), respectively. Although ALT activity in the E9 group showed a slight rebound, it remained below the control level. In contrast, serum AST and ALP activities did not differ significantly among groups (*P* > 0.05), indicating that the hydrolysate had a relatively limited effect on these parameters. Regarding protein metabolism, serum ALB concentrations in the E3 and E6 groups were significantly higher than those in the control group, with increases of 17.52% (*P* = 0.008) and 12.12% (*P* = 0.040), respectively. Concurrently, serum UA concentrations exhibited a significant decreasing trend with increasing supplementation levels, with reductions of 45.52% (*P* = 0.036) and 61.78% (*P* = 0.009) in the E6 and E9 groups, respectively, compared with the control group. Although UREA concentrations did not reach statistical significance (*P* > 0.05), a decreasing tendency was also observed. Regarding lipid metabolism, serum TG, CHO, and LDL-C concentrations did not change significantly among groups (*P* > 0.05). However, HDL-C concentrations showed a linearly increasing trend with increasing hydrolysate supplementation levels (linear *P* = 0.034), reaching the highest value in the E9 group.

**Table 5:** Effects of BSFH with different addition amounts on serum biochemical indices of layer chicks.

Item	Group				SEM	*P*-value		
	Control	E3	E6	E9		ANOVA	Linear	Quadratic
ALT/(U/L)	3.53±0.77^a^	1.77±0.69^b^	1.94±0.57^b^	2.43±0.35^ab^	0.26	0.030	0.184	0.015
AST/(U/L)	259.04±20.70	231.07±24.17	245.39±10.78	252.22±22.87	5.90	0.427	0.914	0.368
ALB/(g/L)	15.01±0.40^b^	17.64±0.80^a^	16.83±0.46^a^	16.07±1.52^ab^	0.37	0.039	0.498	0.034
ALP/(U/L)	2,103.80±207.02	2,300.94±962.95	2,363.82±234.92	2,277.23±605.66	148.11	0.954	0.681	0.838
TBA/(μmol/L)	6.38±2.95	6.32±0.41	5.67±3.44	4.32±1.64	0.65	0.712	0.256	0.482
UREA/(mmol/L)	0.50±0.04	0.48±0.12	0.47±0.12	0.41±0.11	0.03	0.768	0.297	0.559
UA/(μmol/L)	266.74±81.56^a^	234.57±17.88^ab^	145.33±16.72^bc^	101.95±81.57^c^	24.71	0.030	0.002	0.011
TG/(mmol/L)	0.36±0.02	0.36±0.11	0.35±0.05	0.36±0.04	0.02	0.997	0.937	0.982
CHO/(mmol/L)	3.28±0.33	3.02±0.14	3.14±0.11	3.22±0.35	0.07	0.634	0.930	0.505
HDL-C/(mmol/L)	1.93±0.18	2.07±0.16	2.19±0.14	2.37±0.42	0.08	0.258	0.034	0.118
LDL-C/(mmol/L)	1.04±0.27	0.99±0.23	0.96±0.10	0.94±0.29	0.06	0.960	0.557	0.853

### Antioxidant parameters of layer chicks fed BSFH

As shown in Table 6, serum T-SOD activity exhibited an initial increase followed by a decrease with increasing supplementation levels of black soldier fly hydrolysate, with the highest activity observed in the E6 group, and the linear trend was statistically significant (*P* = 0.044). This indicates that black soldier fly hydrolysate, within a certain supplementation range, may enhance the capacity to scavenge superoxide anions. Both GSH-Px and CAT activities peaked in the E3 group, with significant increases of 22.48% (*P* = 0.013) and 52.11% (*P* = 0.008), respectively, compared with the control group. However, with further increases in supplementation level, the activities of both enzymes declined. This finding suggests that relatively low-dose supplementation can effectively activate certain antioxidant enzyme systems, whereas higher doses do not confer additional benefits. Serum MDA content and T-AOC did not differ significantly among groups (*P* *>* 0.05), indicating that the hydrolysate did not exert a pronounced impact on serum lipid peroxidation levels or overall antioxidant status in chicks.

**Table 6:** Effects of Different addition amounts of BSFH on antioxidant indices of layer chicks.

Part	Item	Group				SEM	*P*-value		
		Control	E3	E6	E9		ANOVA	Linear	Quadratic
Serum	T-SOD/(U/mg prot)	205.40±8.67	212.73±21.20	239.14±14.42	234.12±28.63	6.46	0.184	0.044	0.128
	MDA/(μmol/L)	4.37±0.48	4.26±0.95	4.08±0.50	3.69±0.28	0.16	0.844	0.339	0.649
	GSH-Px/(μmol/L)	6.45±0.63^b^	7.90±0.14^a^	6.95±0.85^ab^	6.48±0.31^b^	0.22	0.041	0.689	0.076
	CAT/(U/mL)	9.46±1.12^b^	14.39±2.67^a^	10.41±1.01^b^	10.27±1.63^b^	0.72	0.033	0.823	0.217
	T-AOC/(mM)	0.86±0.09	1.08±0.09	0.98±0.24	0.87±0.05	0.04	0.251	0.897	0.170
Liver	T-SOD/(U/mg prot)	333.30±24.40	345.14±28.05	373.33±15.23	353.30±27.42	7.43	0.302	0.197	0.251
	MDA/(μmol/L)	0.64±0.20	0.63±0.13	0.62±0.10	0.62±0.06	0.03	0.999	0.861	0.985
	GSH-Px/(μmol/L)	7.45±1.23	7.61±0.75	7.92±0.48	7.64±0.76	0.21	0.967	0.989	0.989
	CAT/(U/mL)	530.43±31.16^b^	539.57±22.68^b^	638.91±40.52^a^	577.82±19.05^b^	14.78	0.008	0.064	0.079
	T-AOC/(mM)	0.13±0.03	0.13±0.02	0.14±0.01	0.12±0.01	0.01	0.727	0.707	0.587

In liver tissue, only CAT activity showed a significant difference, being significantly higher in the E6 group than in all other groups (*P* = 0.002), with a linearly increasing trend approaching statistical significance. This observation suggests that supplementation at the 0.6% level may have promoted the capacity of the liver to scavenge hydrogen peroxide. No significant changes were observed in the remaining hepatic indicators across groups (*P* *>* 0.05), reflecting that the hepatic antioxidant system was relatively less affected by this intervention overall.

### Intestinal morphology of layer chicks fed BSFH

The results are presented in Table 7. The effects of BSFH on intestinal morphology exhibited segment specificity. In the duodenum and ileum, no significant differences were observed in villus height, crypt depth, or V/C ratio between the experimental groups and the control group (*P* > 0.05), indicating that the hydrolysate did not exert a pronounced effect on the macrostructure of these two intestinal segments. In contrast, the jejunum, as the primary site for nutrient absorption, exhibited greater structural plasticity, thereby serving as the key intestinal segment through which the protein hydrolysate exerted its modulatory effects. With increasing supplementation levels, jejunal crypt depth exhibited a linearly decreasing trend (linear *P* = 0.038). Although pairwise comparisons did not detect significant differences between any individual groups (all *P* > 0.05), the overall dose-dependent pattern suggests a progressive reduction in crypt depth with BSFH supplementation. Concurrently, the jejunal V/C ratio showed highly significant linear and quadratic increases with increasing supplementation levels (linear *P* = 0.002; quadratic *P* = 0.010), with increases of 6.10% (*P* = 0.459), 17.32% (*P* = 0.053), and 26.34% (*P* = 0.008) in the E3, E6, and E9 groups, respectively, compared with the control group. Villus height did not change significantly among groups, indicating that the increase in V/C ratio was primarily attributable to reduced crypt depth.

**Table 7:** Effects of BSFH with different addition amounts on the intestinal morphology of layer chicks.

Intestinal tissue	Item	Group				SEM	*p-*Value		
		Control	E3	E6	E9		ANOVA	Linear	Quadratic
Duodenum	Villus height/μm	1,627.93±189.27	1,793.56±129.41	1,702.62±167.44	1,722.34±191.16	45.73	0.708	0.660	0.694
	Crypt depth/μm	333.03±27.60	353.16±17.00	323.57±23.03	318.60±51.04	8.91	0.595	0.385	0.559
	V/C	4.03±0.90	5.09±0.48	5.30±0.85	5.45±0.56	0.19	0.828	0.319	0.626
Jejunum	Villus height/μm	1,348.71±247.09	1,447.09±138.27	1,375.78±79.41	1,374.64±90.76	39.45	0.876	0.984	0.848
	Crypt depth/μm	326.89±39.22	333.06±33.88	290.62±54.33	267.55±10.90	12.35	0.193	0.038	0.105
	V/C	4.10±0.38^b^	4.35±0.06^b^	4.81±0.59^ab^	5.18±0.27^a^	0.16	0.033	0.002	0.010
Ileum	Villus height/μm	1,332.89±129.25	1,364.09±26.98	1,378.28±25.69	1,368.36±36.60	17.91	0.868	0.478	0.628
	Crypt depth/μm	278.07±8.68	275.97±49.12	269.00±12.50	248.85±8.62	7.30	0.536	0.155	0.316
	V/C	4.80±0.52	5.07±1.11	5.13±0.27	5.50±0.20	0.17	0.618	0.172	0.411

### Cecal microbiota of layer chicks fed BSFH

As shown in Fig. 1A, the rarefaction curves for all experimental groups rapidly approached a plateau, indicating that the current sequencing depth was sufficient to capture the vast majority of microbial species in the samples, thereby providing a reliable data foundation for subsequent diversity analyses. The results of alpha diversity analysis are presented in Fig. 1C. Regarding species richness, the ACE and Chao indices in all experimental groups were numerically higher than those in the control group; however, these differences did not reach statistical significance (*P* > 0.05). This finding suggests that hydrolysate supplementation may have exerted a modest positive effect on the number of microbial species in the intestine. In terms of species diversity, no significant differences were observed in the Shannon or Simpson indices among the groups, indicating that hydrolysate supplementation did not markedly alter community evenness, and all groups maintained a high level of microbial diversity. The PCoA based on Bray-Curtis distances (Fig. 1B) revealed that samples from different treatment groups were intermingled in the principal coordinate space without forming distinct clusters (*P* > 0.05). This further demonstrates that, despite the trend toward increased richness indices, the addition of the hydrolysate did not significantly alter the overall structural composition of the intestinal microbial community.

**Fig. 1:**
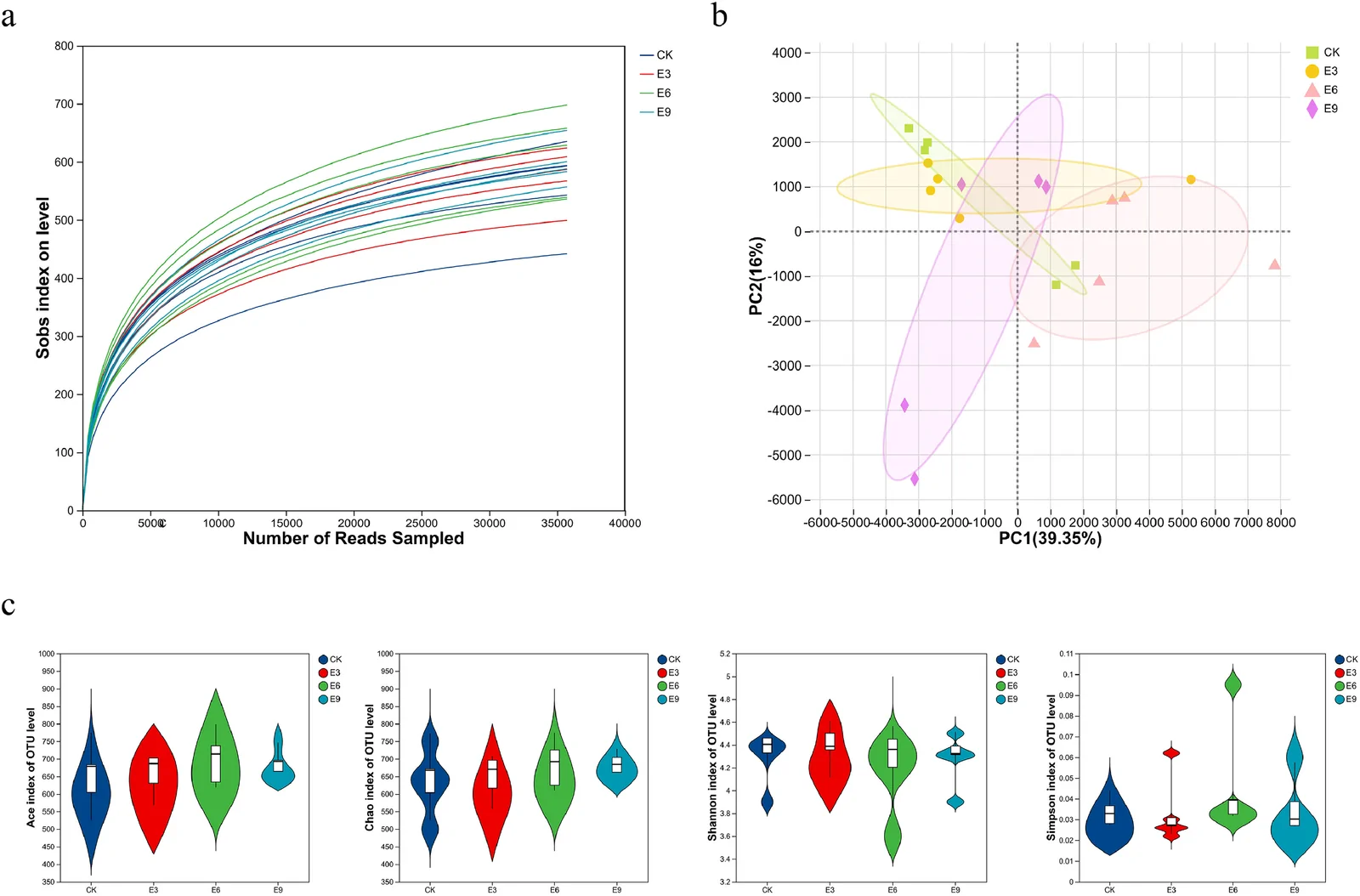
The effect of dietary addition of hydrolyzed products on the Alpha and Beta diversity of the cecal microbial community in chickens. Five cecal content samples per group (n = 5). (a) Dilution curve. (b) Principal coordinate analysis (PCoA) based on Bray-Curtis distance. (c) Alpha diversity at the genus level.

As shown in Fig. 2, at the genus level, the 14 most abundant genera were Bacteroides, Rikenellaceae_RC9_gut_group, Mediterraneibacter, Prevotellaceae_UCG-001, Parabacteroides, unclassified_o__Bacteroidales, norank_o__Clostridia_UCG-014, norank_f__Prevotellaceae, Olsenella, unclassified_f__Lachnospiraceae, norank_f__Muribaculaceae, Phascolarctobacterium, Fusobacterium, and Faecalibacterium. At the genus level, Bacteroides, Rikenellaceae_RC9_gut_group, and Mediterraneibacter remained dominant across all experimental groups, indicating a relatively stable overall microbial community structure. However, specific changes in abundance were observed in several key genera with distinct metabolic functions. In the present experiment, compared with the control group, the relative abundance of Fusobacterium in the E3, E6, and E9 groups increased by 1.37%, 5.05% (P = 0.011), and 0.12%, respectively, with the most pronounced enrichment observed at the 0.6% supplementation level. However, the genus includes both commensal and pathogenic species, such as the opportunistic pathogen Fusobacterium necrophorum (Aguiar et al., 2020). The species-level composition and functional significance of this genus warrant further investigation.

**Fig. 2:**
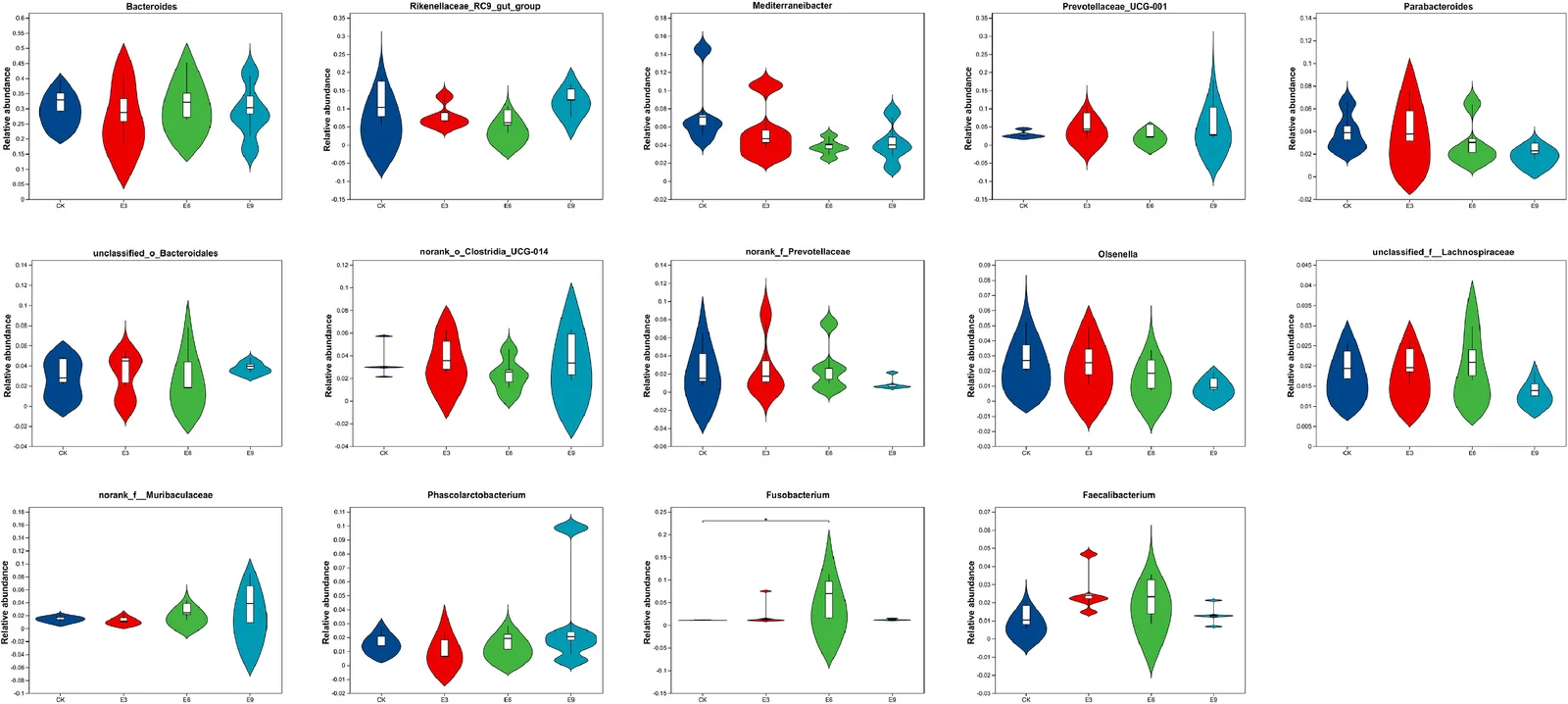
Violin plots of the top 14 genera (genus level, relative abundance) illustrating intergroup differences in taxonomic abundance following dietary hydrolysate addition. Cecal contents from 5 chickens per treatment group (n = 5).

## Discussion

The present study demonstrated that dietary supplementation with 0.3%∼0.9% BSFH significantly increased ADG and improved F/G in layer chicks, without significantly affecting ADFI. These findings are generally consistent with previous reports on the application of black soldier fly products in poultry (Nayohan et al., 2022; Wamai et al., 2023). BSFH is produced by an enzymatic hydrolysis process that breaks down black soldier fly larval proteins into small molecular peptides and free amino acids, and this pre-digested form substantially enhances its bioavailability. Compared with intact proteins, small molecular peptides and free amino acids bypass the complex digestive cascade involving pepsin and pancreatic proteases, thereby reducing the burden on the incompletely developed digestive system of chicks (Abdollahi et al., 2017). This efficient absorption directly promotes systemic protein synthesis and improves nitrogen utilization efficiency, which explains the observed growth improvement in the absence of increased feed intake (Lu et al., 2022b). The intestine is the primary site of nutrient digestion and absorption, and villus height, crypt depth, and the V/C ratio are key morphological indicators of intestinal structural health and absorptive function (Asmaz and Seyidoğlu, 2022). Regarding intestinal morphology, BSFH supplementation did not significantly affect villus height, crypt depth, or the V/C ratio in the duodenum or ileum. In the jejunum, however, it significantly reduced crypt depth and increased the V/C ratio without altering villus height. This indicates that BSFH acts principally on the jejunum, the main site of nutrient absorption, by reducing crypt depth to optimize intestinal structure and lower the energy cost of epithelial turnover, thereby enhancing nutrient absorption efficiency. Notably, despite these improvements in intestinal morphology, 16S rRNA gene sequencing revealed no significant differences in cecal microbial diversity between the experimental and control groups. In contrast, He et al. (2021) reported that intact BSFLM remodeled the cecal microbiota, whereas BSFH in the present study, although improving jejunal morphology, did not significantly alter cecal community structure. The two differ mainly in protein form: hydrolysis cleaves the intact larval proteins into low-molecular-weight peptides, which are absorbed proximally and leave little fermentable substrate for the hindgut. BSFH therefore acts mainly through host-mediated pathways, yielding only modest, taxon-specific cecal shifts rather than broad restructuring.

The patterns of change observed in serum biochemical parameters provide critical evidence for evaluating the effects of BSFH on liver function and systemic metabolic homeostasis in chicks. In the present study, dietary supplementation with BSFH resulted in significantly decreased serum ALT and UA levels, alongside increased ALB concentrations. Alanine aminotransferase, a commonly used marker of hepatocellular injury (Huff et al., 1986), showed reduced serum activity in the E3 and E6 groups, suggesting that BSFH supplementation did not induce hepatocellular damage in these chicks. These findings are consistent with previous studies on the application of black soldier fly products in poultry (Attivi et al., 2020; Chang et al., 2025). Albumin, synthesized by the liver, is an important indicator of hepatic synthetic capacity and overall nutritional status (Zhuang et al., 2019). In the present study, BSFH supplementation resulted in significantly increased serum albumin concentrations in the E3 and E6 groups, indicating adequate nitrogen availability for hepatic protein synthesis. Research by Kryuchkov and Nikisov (2025) demonstrated that dietary inclusion of defatted black soldier fly meal significantly increased serum albumin concentrations in growing layer hens; similarly, Rytlewski et al. (2025) observed elevated serum total protein and albumin levels in pheasant broilers fed BSFL. These previous findings, together with the increased ALB levels observed in the present study, strongly indicate that BSFH provides adequate substrates for hepatic synthesis, effectively supporting systemic protein anabolism. The observed reduction in serum UA levels directly reflects improved nitrogen metabolism efficiency. Nayohan et al. (2022) and Fikri et al. (2024) reported that black soldier fly supplementation reduced serum UA levels in chickens, suggesting that the high-quality, readily digestible protein provided by black soldier fly products minimizes excessive endogenous protein catabolism, thereby decreasing purine nucleotide turnover and the subsequent production of uric acid. This not only represents enhanced nitrogen utilization efficiency but also implies a reduced excretory burden on the kidneys.

Enzymatic hydrolysis of black soldier fly protein effectively releases bioactive small molecular peptides, which exhibit excellent antioxidant capacity due to their specific amino acid residues (Batish et al., 2020; Zhu et al., 2020; Lu et al., 2022a). Dietary supplementation with these prepared black soldier fly enzymatic hydrolysate peptides in poultry, particularly in chicks during their rapid growth phase when they are susceptible to oxidative stress, can significantly improve their antioxidant status (Nawab et al., 2018). The levels of T-SOD, MDA, GSH-Px, CAT, and T-AOC in the serum and liver of chicks are core indicators for evaluating antioxidant status, comprehensively reflecting the potential capacity of chicks to resist oxidative damage (Li et al., 2020). In the present study, dietary supplementation with BSFH specifically enhanced the activity of certain antioxidant enzymes in chicks. Serum T-SOD activity exhibited a linearly increasing trend with increasing BSFH supplementation levels, reaching its maximum value in the E6 group. Furthermore, GSH-Px and CAT activities were significantly higher in the E3 group compared with the control group, suggesting that relatively low-dose BSFH supplementation effectively activates components of the antioxidant enzyme system, whereas activities declined back to near-control levels at the highest supplementation level (0.9%). This biphasic dose-response pattern is consistent with the bell-shaped dose-response model for antioxidant enzymes (McCord, 2008), in which low doses activate endogenous antioxidant defenses through the nuclear factor erythroid 2-related factor 2 (**Nrf2**) signaling while high doses may suppress enzyme activity via negative feedback. These observations suggest that an optimal dose window exists for BSFH supplementation in layer chicks. Studies have demonstrated that the small molecular peptides derived from BSFH exert antioxidant effects by mimicking the active center of superoxide dismutase or activating Nrf2 signaling pathway (Mouithys-Mickalad et al., 2020; Riolo et al., 2023). The Nrf2 pathway is a central regulator of antioxidant enzyme expression in vertebrates, including poultry species; activation of this pathway by bioactive compounds has been demonstrated in broiler chickens, where dietary supplementation with antioxidants upregulated hepatic expression of Nrf2, heme oxygenase-1 (HO-1), and SOD1, accompanied by increased activities of CAT, GSH-Px, and SOD (Zhang et al., 2021). These findings have been corroborated in feline and beagle models, where dietary supplementation with BSFH significantly enhanced serum antioxidant capacity, a result that aligns closely with the observations of the present study (Li et al., 2024; Wei et al., 2024). In liver tissue, CAT activity was significantly higher in the E6 group than in all other groups, suggesting that supplementation at the 0.6% level may have specifically enhanced the hepatic capacity to scavenge hydrogen peroxide, although no significant changes were observed in other hepatic antioxidant indicators.

Collectively, the results of this study indicate that appropriate dietary supplementation with BSFH in layer chicks not only effectively promotes growth and improves feed efficiency but may also exert positive effects on immune organ development and comprehensively enhance chick health status by optimizing physiological metabolism, strengthening antioxidant capacity, and improving intestinal health and microbial communities. These findings support BSFH as a sustainable and functional protein source that can partially replace traditional protein ingredients, thereby providing an environmentally friendly and economically efficient feed solution for the poultry industry. Future research should further investigate the underlying mechanisms of BSFH on intestinal health, antioxidant capacity, and specific immune responses, as well as optimize its optimal inclusion levels in diets for poultry at different growth stages.

## Conclusion

In conclusion, this study demonstrated that dietary supplementation with 0.3%–0.9% BSFH dose-dependently improved growth performance and feed conversion efficiency in layer chicks: ADG was significantly increased in the E6 and E9 groups and F/G was significantly reduced in the E9 group, without affecting feed intake; meanwhile, the activity of specific serum and hepatic antioxidant enzymes was enhanced and jejunal morphology was improved. These effects suggest that BSFH acts by improving nutrient utilization efficiency and strengthening antioxidant defense, supporting its potential as a functional feed additive. Nevertheless, immune organ indices and cecal microbial diversity were not significantly affected, and the beneficial effects were limited to specific parameters. From a practical perspective, the unpurified hydrolysate used in this study may pose a certain storage risk, and its economic feasibility relative to conventional soybean meal remains to be assessed. Future research should evaluate the storage stability of BSFH-supplemented feeds and elucidate the underlying mechanisms to determine whether BSFH can serve as a practically viable and economically sustainable feed ingredient for the poultry industry.

Declaration of Generative AI and AI‑Assisted Technologies in the Writing Process: The authors used DeepL and DeepSeek to enhance readability and language refinement while preparing this work. After using these tools, the authors reviewed and edited the content as necessary and take full responsibility for the content of the publication.

## CRediT authorship contribution statement

**Xinhao Zhou:** Conceptualization, Data curation, Formal analysis, Investigation, Writing – original draft. **Wenye Zhang:** Data curation, Formal analysis, Investigation, Methodology. **Shuaichen Yao:** Data curation, Investigation, Validation, Visualization. **Yaxin Pei:** Conceptualization, Supervision, Project administration. **Jiran Zhang:** Methodology, Supervision, Writing – review & editing. **Xiaojun Feng:** Resources, Supervision, Writing – review & editing. **Sen Yang:** Conceptualization, Supervision, Funding acquisition, Writing – review & editing.

## Disclosures

The authors declare the following financial interests/personal relationships which may be considered as potential competing interests:

Sen YANG reports financial support was provided by Henan Provincial Department of Science and Technology. If there are other authors, they declare that they have no known competing financial interests or personal relationships that could have appeared to influence the work reported in this paper.
